# Examining the impact of a symptom assessment application on patient-physician interaction among self-referred walk-in patients in the emergency department (AKUSYM): study protocol for a multi-center, randomized controlled, parallel-group superiority trial

**DOI:** 10.1186/s13063-022-06688-w

**Published:** 2022-09-20

**Authors:** Hendrik Napierala, Marvin Kopka, Maria B. Altendorf, Myrto Bolanaki, Konrad Schmidt, Sophie K. Piper, Christoph Heintze, Martin Möckel, Felix Balzer, Anna Slagman, Malte L. Schmieding

**Affiliations:** 1 Charité – Universitätsmedizin Berlin, corporate member of Freie Universität Berlin and Humboldt-Universität zu Berlin, Institute of General Practice and Family Medicine, Charitéplatz 1, 10117 Berlin, Germany; 2 Charité – Universitätsmedizin Berlin, corporate member of Freie Universität Berlin and Humboldt-Universität zu Berlin, Institute of Medical Informatics, Charitéplatz 1, 10117 Berlin, Germany; 3 Cognitive Psychology and Ergonomics, Department of Psychology and Ergonomics (IPA), Technische Universität Berlin, Straße des 17. Juni 135, 10623 Berlin, Germany; 4 Charité – Universitätsmedizin Berlin, corporate member of Freie Universität Berlin and Humboldt-Universität zu Berlin, Emergency and Acute Medicine and Health Services Research in Emergency Medicine (CVK, CCM), Charitéplatz 1, 10117 Berlin, Germany; 5 Jena University Hospital, Institute of General Practice and Family Medicine, Bachstr. 18, 07743 Jena, Germany; 6 Charité – Universitätsmedizin Berlin, corporate member of Freie Universität Berlin and Humboldt-Universität zu Berlin, Institute of Biometry and Clinical Epidemiology, Charitéplatz 1, 10117 Berlin, Germany; 7 Berlin Institute of Health at Charité – Universitätsmedizin Berlin, Charitéplatz 1, 10117 Berlin, Germany; 8 docport Services GmbH, Tußmannstr. 75, 40477 Düsseldorf, Germany

**Keywords:** Symptom assessment application, Symptom checker, Randomized controlled trial, Emergency medicine, Patient-physician interaction, Clinical decision support, Consumer health IT, Online health information

## Abstract

**Background:**

Due to the increasing use of online health information, symptom checkers have been developed to provide an individualized assessment of health complaints and provide potential diagnoses and an urgency estimation. It is assumed that they support patient empowerment and have a positive impact on patient-physician interaction and satisfaction with care. Particularly in the emergency department (ED), symptom checkers could be integrated to bridge waiting times in the ED, and patients as well as physicians could take advantage of potential positive effects. Our study therefore aims to assess the impact of symptom assessment application (SAA) usage compared to no SAA usage on the patient-physician interaction in self-referred walk-in patients in the ED population.

**Methods:**

In this multi-center, 1:1 randomized, controlled, parallel-group superiority trial, 440 self-referred adult walk-in patients with a non-urgent triage category will be recruited in three EDs in Berlin. Eligible participants in the intervention group will use a SAA directly after initial triage. The control group receives standard care without using a SAA. The primary endpoint is patients’ satisfaction with the patient-physician interaction assessed by the Patient Satisfaction Questionnaire.

**Discussion:**

The results of this trial could influence the implementation of SAA into acute care to improve the satisfaction with the patient-physician interaction.

**Trial registration:**

German Clinical Trials Registry DRKS00028598. Registered on 25.03.2022

## Administrative information

Note: the numbers in curly brackets in this protocol refer to SPIRIT checklist item numbers. The order of the items has been modified to group similar items (see http://www.equator-network.org/reporting-guidelines/spirit-2013-statement-defining-standard-protocol-items-for-clinical-trials/).

**Table:** 

Title {1}	Examining the impact of a symptom assessment application on patient-physician-interaction among self-referred walk-in patients in the emergency department (AKUSYM): study protocol for a multi-center, randomized controlled, parallel-group superiority trial
Trial registration {2a and 2b}.	Registered at German Clinical Trials Register: DRKS00028598
Protocol version {3}	2022-05-31 Version 1.0
Funding {4}	Funded by a grant from the German Ministry of Health (Funding number 2521TEL500).
Author details {5a}	Hendrik Napierala^1^*, Marvin Kopka^2,3^*, Maria B. Altendorf^4^, Myrto Bolanaki^4^, Konrad Schmidt^1,6^, Sophie K. Piper^2,7,8^, Christoph Heintze^1^, Martin Möckel^4^, Felix Balzer^2^, Anna Slagman^4^*, Malte L. Schmieding^2,5^*^#^ 1 Charité – Universitätsmedizin Berlin, corporate member of Freie Universität Berlin and Humboldt-Universität zu Berlin, Institute of General Practice and Family Medicine, Charitéplatz 1, 10117 Berlin, Germany 2 Charité – Universitätsmedizin Berlin, corporate member of Freie Universität Berlin and Humboldt-Universität zu Berlin, Institute of Medical Informatics, Charitéplatz 1, 10117 Berlin, Germany 3 Cognitive Psychology and Ergonomics, Department of Psychology and Ergonomics (IPA), Technische Universität Berlin, Straße des 17. Juni 135, 10623 Berlin, Germany 4. Charité – Universitätsmedizin Berlin, corporate member of Freie Universität Berlin and Humboldt-Universität zu Berlin, Emergency and Acute Medicine and Health Services Research in Emergency Medicine (CVK, CCM), Charitéplatz 1, 10117 Berlin, Germany 5. docport Services GmbH, Tußmannstr. 75, 40477 Düsseldorf, Germany 6. Institute of General Practice and Family Medicine, D-07743 Jena University Hospital, Germany 7. Charité – Universitätsmedizin Berlin, corporate member of Freie Universität Berlin and Humboldt-Universität zu Berlin, Institute of Biometry and Clinical Epidemiology, Charitéplatz 1, 10117 Berlin, Germany 8. Berlin Institute of Health at Charité – Universitätsmedizin Berlin, Charitéplatz 1, 10117 Berlin, Germany Chief Investigator: FB. Co-Lead: MLS. Study concept and design: HN, MK, AS, MLS. Methodology: HN, SKP, AS. Authors of study protocol: HN, MK, MBA, MLS. Study supervision: CH, FB, MM. Funding: MK, FB, AS, KS, MLS. Critical input: MB, KS, CH, SKP, MM. All authors contributed to refinement of the study protocol and approved the final manuscript. *Authors contributed equally ^#^Corresponding author
Name and contact information for the trial sponsor {5b}	Trial sponsor: Institute of Medical Informatics Address: Charité - Universitätsmedizin Berlin, Charitéplatz 1, 10117 Berlin, Germany Contact: Prof. Dr. Dr. Felix Balzer Telephone: +49 30 450 570 425 E-mail: sekretariat-balzer@charite.de
Role of sponsor {5c}	The sponsor is responsible for providing the necessary structural, economical and personnel resources to ensure the study participants safety and to carry out the study. The Principal Investigator and the research team (authors) are responsible for the study design, collection, management, analysis, and interpretation of data and writing of the report or publication. The funding source had no role in the design of this study and will not have any role during its execution, analyses, interpretation of the data, or decision to submit results.

## Introduction

### Background and rationale {6a}

According to a representative survey among the German population, 20% of the participants named the internet as their primary source of health information [1]. Especially patients seeking emergency care services use online information to self-assess their symptoms and search for potential diagnoses prior to their visit [2–4]. In recent years, special applications have been developed for this purpose: symptom assessment applications (SAA) are smartphone- or web-based applications for laypersons providing an individualized assessment of the entered health complaints by providing suggestions on likely diagnoses and a categorization of their treatment urgency. In Germany, the number of users of these systems has risen to 13% in 2020 [5]. Such apps provide a convenient option for patients to obtain information in preparation for contact with the physician. SAA could empower patients to provide the physician with more specific information during anamnesis and support them to prepare their own questions [6]. This, in turn, could have a positive impact on patient-physician interaction. While studies on SAA accuracy of assessment already exist [7–11], current research is insufficient to evaluate the effects of SAA on their users, as well as their received care and the treating physicians. Thus, it is unclear whether the individual value of symptom checkers translates into a meaningful and quantifiable improvement of care delivery, patient satisfaction, and an improvement of the physician-patient relationship. Two observational studies showed positive effects of using Google as a source of online health information on the perceived quality of received care and patient-physician interaction [3, 4], but a randomized controlled trial could not confirm this effect [2]. To this end, it is possible that reported positive effects are not attributable to the information search per se, but rather to the fact that patients who obtain health information on their own are also better able to seek care. In contrast to internet search-based online health information seeking, symptom checkers guide their users through the self-assessment process and provide more custom-tailored information, for example, on plausible diagnoses and appropriate therapies. Thus, SAA recommendations might constitute a fruitful basis for the patient’s interaction with the treating physician. Particularly in the emergency department (ED), with longer waiting times, symptom checkers could be integrated to bridge waiting times in the ED and take advantage of potential positive effects. Our study therefore aims to assess the impact of SAA usage as a special type of online health information on the patient-physician interaction and care delivery in the ED.

### Objectives {7}

#### Primary objective

To investigate the impact on patients’ satisfaction with the patient-physician interaction of a SAA prior to the first physician encounter among self-referred walk-in patients in the ED setting.

#### Secondary objectives

1. To investigate the impact of a SAA prior to the first physician encounter among self-referred walk-in patients in the ED setting on satisfaction with the received care at the end of the ED visit.
2. To investigate the impact of a SAA prior to the first physician encounter among self-referred walk-in patients in the ED setting on anxiety of participants.
3. To investigate the impact of a SAA prior to the first physician encounter among self-referred walk-in patients in the ED setting on physicians’ satisfaction with the patient-physician interaction after the patient-physician encounter.

#### Other objectives

4. To examine the level of trust in the SAA after using it.
5. To assess the usability and usefulness of the app-based self-assessment as rated by the participants.
6. To translate and validate a German version of the mHealth App Usability Questionnaire (MAUQ).
7. To assess participants’ decisional certainty in their stand-alone and app-supported triage recommendations.
8. To assess diagnostic and triage accuracy of the app-based self-assessment.
9. To assess the prognostic value of the triage to predict hospitalization and other clinical endpoints.
10. To compare patients’ and physicians’ appraisals of patients’ eHealth-literacy, and the usability and usefulness of the SAA for patients.

### Trial design {8}

The study is a parallel-group, multi-center superiority trial with a randomization allocation ratio of 1:1 to either using a SAA before the physician encounter or physician encounter only (standard care) among non-urgent, self-referred walk-in patients in the ED.

## Methods: participants, interventions, and outcomes

### Study setting {9}

The study is a local, multi-center ED-based study with three study sites located in Berlin, Germany. Two of the study sites are EDs of a large tertiary care hospital (Charité – Universitätsmedizin Berlin, Campus Mitte/Campus Virchow-Klinikum). One site is an emergency practice of the association of statutory health insurance physicians attached to the ED of a local hospital (Jüdisches Krankenhaus Berlin).

### Eligibility criteria {10}

#### Inclusion criteria

Patients eligible for the trial must comply with all of the following at randomization:

1. Age ≥ 18
2. Treatment urgency assessed with the Manchester triage system: categorized as yellow (urgent, 30 min.), green (standard, 90 min.), or blue (non-urgent, 120 min.)
3. Self-referred walk-in
4. Adequate German or English language skills
5. The patient is able to provide informed consent.

#### Exclusion criteria

1. Patient treated without waiting time
2. Cause of complaint known according to patient
3. Patient requiring isolation
4. Patient who would not be able to use a tablet according to the assessment of the study personnel on site or according to their own judgment
5. Patient consulted a symptom checker app for the current complaints prior to seeking health care

### Who will take informed consent? {26a}

Participants eligible for inclusion will be invited by study personnel to give written and verbal informed consent directly after the initial triage. The participant will be handed a copy of the Human Research Ethics Committee/Institutional Review Board-approved informed consent form. The right of a participant to refuse participation without giving reasons will be respected. The participant is free to withdraw from the trial at any time without giving reasons and without prejudicing the participant’s further treatment.

All site investigators and delegates will be trained and competent to participate according to the ethically approved protocol, principles of Good Clinical Practice (GCP), and the Declaration of Helsinki.

### Additional consent provisions for collection and use of participant data and biological specimens {26b}

Participants will be asked if they wish to be contacted for a separate study. This study consists of approximately 15-20 semi-structured qualitative interviews with patients from the intervention group. In addition, we will perform two focus groups with health care professionals (treating physicians and nurses, 6-10 per group) who treated study participants in the acute care setting.

There will be no collection of biological specimens beyond standard care.

## Interventions

### Explanation for the choice of comparators {6b}

Patients in the control group proceed as usual and only complete questionnaires at the beginning of their waiting period and at the end of the ED visit. Otherwise, they wait without further intervention and receive standard care to ensure that the experimental intervention can be compared to the regular ED procedure. In extraordinary circumstances, patients can be contacted via telephone for up to 72 h to reduce loss-to-follow-up.

### Intervention description {11a}

Eligible patients randomized to the intervention group will use the SAA Ada (© 2021 Ada Health GmbH: www.ada.com). Patients will be asked to use Ada once to assess their symptoms on a study tablet—which will be provided by the study nurses—while waiting in the ED before their first consultation with a physician. Once completed, they receive a list of possible diagnoses and an estimation of their complaints’ urgency from the SAA. The assessment and recommendations are printed by the study personnel and will be provided to the treating physician before the ED consultation. Thus, the intervention is the same for all patients, but the received advice is tailored to their prior responses when using the symptom checker.

### Criteria for discontinuing or modifying allocated interventions {11b}

Patients will use the SAA with an estimated mean completion time of around 8 min. If patients decide to not finish the assessment, e.g. withdrawal of consent, they will stop using the app and are considered according to the randomized group in the intention to treat analysis but excluded from per protocol analysis.

### Strategies to improve adherence to interventions {11c}

Patients will be informed about the study and the SAA and asked to participate. Since the intervention is implemented once only, no further measures for adherence improvement regarding the intervention are planned. Participants will receive small financial incentives (vouchers) if they complete the study, this also includes all study questionnaires.

### Relevant concomitant care permitted or prohibited during the trial {11d}

No interventions are prohibited. However, the use of other SAA or online health information while waiting is discouraged.

### Provisions for post-trial care {30}

Patient harm from trial participation is not anticipated but all patients are encouraged to re-contact the ED or an ambulatory health care provider in case of deterioration of symptoms as part of standard care. Furthermore, patients are encouraged to contact the principal investigator of the study in case of any study-related questions or problems that might occur.

### Outcomes {12}

#### Primary outcome

The primary outcome is the participants’ satisfaction with the patient-physician-interaction as measured by the the Patient Satisfaction Questionnaire (PSQ) [12]. The intervention is considered successful if we find a mean difference in means of at least 5 points (Scale 0-100) in the intervention group compared to the control group after the first doctor’s contact before discharge, in exceptional cases by telephone within 72h.

#### Secondary outcomes

The secondary outcomes are as follows:

- Patients’ satisfaction with the patient-physician interaction is measured using the ZUF-8 [13] (German version of the Client Satisfaction Questionnaire, CSQ-8 [14]). We expect a 1-point mean increase (scale 8–32) between the intervention group and the control group after patients have been treated (at the end of the ED visit before discharge, in exceptional cases by telephone within 72h). In contrast to the PSQ, the CSQ measures satisfaction with the provided health care services.
- Anxiety is measured using a visual analog scale (VAS-A) [15] at baseline, after the symptom assessment, and at the end of the ED visit. We expect that the potential increase in anxiety after symptom assessment from the baseline value will not be higher than 5 points in the intervention group and that the proportion of patients leaving the ED with higher anxiety values than at baseline will not be higher in the intervention group than in the control group.
- Physicians’ satisfaction with the patient-physician-interaction as measured by the mean score of the physician version of the Patient Satisfaction Questionnaire (PSQ, physician version) [12]. The intervention regarding this endpoint is considered successful if we find a mean difference in means of at least 5 points (scale 0–100) in the intervention group compared to the control group after patients have been treated.

#### Other outcomes

Further, we want to assess additional outcomes relating to the SAA. Most of these outcomes are measured in patients of the intervention group exclusively:

- Certainty in participants’ self-triage decision is measured with a visual analog scale. We consider a 5-point mean difference from baseline to after symptom assessment to be a relevant difference in the intervention group.
- Trust in the symptom checker is measured using a two-item Likert scale.
- Trust in the physician is measured using the trust scale of the Cologne Patient Questionnaire (CPQ) [16] in both groups.
- Usability and usefulness is measured using the System Usability Scale (SUS) [17], the mHealth App Usability Questionnaire (MAUQ) [18], and a self-developed questionnaire for the intervention group only.
- Diagnostic accuracy is measured by comparing the recommendations of the SAA with diagnoses given in the ED, after discharge and by an independent, external, expert panel blinded to the recommendation given by the app.
- Triage accuracy is measured by comparing the recommendation of the SAA with the MTS, triage appraisal of the treating physician in the ED, and by an independent, external, expert panel blinded to the recommendation given by the app for cases where discrepancies occurred.

### Participant timeline {13}

**Fig. 1:**
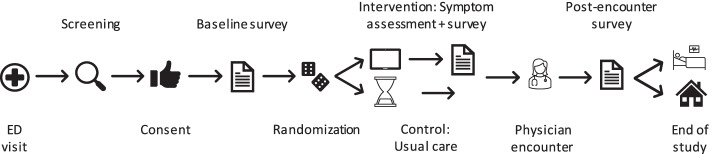
Schematic illustration of a participant's journey through the 2-group parallel randomized controlled trial's phases of enrollment, allocation and data collection at multiple points in time during the visit

Study subjects participate in the study for the duration from admission to the ED until discharge (home or hospitalization). The participant timeline is illustrated in Fig. 1, while Table 1 provides the schedule of events.

**Table 1:** Schedule of events

Timepoint	Study period						
	Screening	Baseline	Treatment	Follow-up			
	***−t***_***1***_	0	***t***_***1***_ ***(after randomization)***	***t***_***2***_ ***(after symptom assessment)***	***t***_***3***_ ***(physician ecnounter)***	***t***_***4***_ ***(after physician encounter, up to 72h)***	***t***_***5***_ ***(post-discharge)***
**Eligibility**							
**Eligibility screen**	X						
**Informed consent**	X						
**Interventions:**							
**Randomization**		X					
***Symptom assessment***			X		X		
***Usual care***					X		
**Assessments**							
**Baseline variables**		X	X				
**Demographics**	X	X		X^a^		X^a^	
***Primary outcomes***						X	
***Secondary outcomes***				X		X	
***Routine clinical data***							X

^a^If ED flow prohibits patients from completing the survey at the intended timepoint

### Sample size {14}

In the literature, a mean PSQ-5 score of 81 and a standard deviation of 14–17 can be found [13]. Assuming a clinically relevant difference of 5 points this amounts to a standardized mean difference of around 0.3.

For a fixed sample size design, the sample size required to achieve a power of 1-β = 0.80 for the two-sample *t*-test assuming equal variances at level *α* = 0.05 (two-sided) under these assumptions amounts to 2 × 176 = 352 patients needed (nQuery Advisor®, version 7.0). The drop-out rate is expected to be medium-sized (20%) due to organizational matters regarding patient flow in the ED. However, through guidance by the study personnel and small financial incentives attrition rates are tried to be kept down. Nevertheless, a potential dilution of the treatment effect due to drop-outs is considered. It is assumed that this can be compensated by additional 20 % of patients to be randomized, and therefore the total sample size required for a fixed sample size design amounts to *n* = 440 patients (220 patients per group).

### Recruitment {15}

Study nurses will approach patients in the waiting room of each study site to ask for participation in the study. Student assistants will assist study nurses in detecting and recruiting patients from the waiting rooms. Data collection is set to a time period of 6 months and the targeted sample size of 440 patients visiting Charité’s EDs in Berlin-Mitte and Berlin-Wedding (CCM and CVK), as well as Jüdisches Krankenhaus Berlin’s emergency practice of the association of statutory health insurance physicians (KV-Bereitschaftspraxis). It is assumed that it is realistic to reach the targeted numbers of patients since the number of patients presenting in the ED within the timeframe of 6 months is significantly higher than the number of participants needed for this study. Around 40,000 patients with the respective MTS categories (3 to 5) visited the EDs in a 6-month period in recent years (unpublished data).

## Assignment of interventions: allocation

### Sequence generation {16a}

The allocation sequence will be generated by the Institute of Medical Informatics, applying a permuted block design with random blocks stratified by study center using computer-generated random numbers. The block size will be concealed until the primary endpoint will be analyzed. Throughout the study, the randomization will be conducted by the Institute of Medical Informatics. The randomization list remains with the Institute of Medical Informatics for the whole duration of the study. Thus, randomization will be conducted without any influence of the principal investigator, study personnel, or physicians.

### Concealment mechanism {16b}

In every study site, sealed opaque envelopes with printed randomization numbers will be available. For each randomization number, the study group can be found inside the envelopes.

### Implementation {16c}

The allocation sequence will be generated by the Institute of Medical Informatics. The study nurse will open the envelope and will find the treatment condition for the randomized patient. Then, the study nurse will provide patients with information about treatment allocation.

## Assignment of interventions: blinding

### Who will be blinded {17a}

Due to the nature of the intervention, neither participants nor staff can be blinded to allocation.

Data analysts will be blinded for analysis of primary outcomes. Data analysts cannot be blinded for analysis of all outcome measures as data for some secondary and other outcome measures are specific to the intervention, e.g. measures on the usability of the symptom assessment application.

### Procedure for unblinding if needed {17b}

This is an open-label trial, therefore unblinding is not necessary.

## Data collection and management

### Plans for assessment and collection of outcomes {18a}

All clinical study data will be registered in a REDCap database (electronic data collection form for clinical studies running on internal servers at Charité – Universitätsmedizin Berlin). The study timeline can be found in Fig. 1. To ensure that ED flow is not compromised through the study, parts of the baseline survey have been flexibilized.

- Baseline data:
  - Sociodemographical data (e.g., age, sex, level of education)
  - eHealth Literacy (eHEALS) [19]
  - Self-reported health (MEHM) [20]
  - Health anxiety (Item 1 of the German version of the Whiteley-Index WI-d, modified to Likert-scale) [21]
  - Technical affinity (ATI-S) [22]
  - Self-efficacy (ASKU) [23]
  - Anxiety (VAS-A) [15]
  - Patient’s stand-alone triage appraisal
- Symptom assessment data will be collected through the Ada Health app
  - An automatically in-app generated report will be downloaded and saved
- Questionnaires pre-symptom assessment, after the symptom assessment (Ada), and after the physician encounter will be directly entered into the RedCap database by the participants. To reduce loss-to-follow-up, the survey after the physician encounter can be filled out up to 72h after the contact (e.g. on the ward or via telephone) if extraordinary circumstances prohibit the patient from taking the survey at the intended timepoint. Questionnaires used in the study are:
  - PSQ [12]
  - ZUF-8 [13]
  - VAS-A [15]
  - Trust scale of the CPQ [16]
  - System Usability Scale (SUS) (only for the intervention group) [17]
  - mHealth App Usability Questionnaire (MAUQ) (only for the intervention group) [18]
- Treating physicians fill out a paper-based survey after the first-patient encounter including the following items:
  - Physician version of the PSQ [12]
  - Triage appraisal
- Routine clinical follow-up data after the patient-physician encounter will be collected by a study nurse and directly entered into the RedCap database.
  - ICD-10 codes
  - Assigned triage level (Manchester Triage System, MTS)
  - Discharge type
  - Length of hospital stay
  - Duration of waiting time

Patients not willing to participate in the trial will be offered to complete an anonymized version of the baseline survey in a paper-based form.

Data collection forms will be made available upon reasonable request by third, academic partners.

### Plans to promote participant retention and complete follow-up {18b}

The study period for each patient ends after discharge from the ED, not taking clinical record data into account. Therefore, no additional follow-up is necessary, where loss-to-follow-up may occur. Flexibility of assessments shall also improve retention. In addition, there are small financial incentives (vouchers) that will be handed out for completing all surveys up to t3 (Table 1).

### Data management {19}

A data management and data protection plan was reviewed and approved by the clinical trial offices (an independent body) of the Charité – Universitätsmedizin Berlin. All data will be password protected on a server run by the Charité – Universitätsmedizin Berlin. Primary data, such as patient-reported outcomes, will be pseudonymized. Clinical data will be extracted from the electronic documentation system of the hospital into electronic case report forms (eCRFs). All extracted data is pseudonymized and will allow subsequent data linkage with patient-reported data from surveys. Range plausibility checks will be applied for data entry. Patients will answer questionnaires on tablets directly imported in RedCap.

### Confidentiality {27}

Only the principal investigator and authorized study members will be able to access the trial data at participating study sites. Recruitment logs will be kept at the local sites in locked cabinets. Pseudonymized data will be archived for 10 years.

### Plans for collection, laboratory evaluation, and storage of biological specimens for genetic or molecular analysis in this trial/future use {33}

Not applicable—see above (26b); there will be no biological specimens collected.

## Statistical methods

### Statistical methods for primary and secondary outcomes {20a}

R 4.0.2 or higher (R Core Team, 2022) and SAS 9.4 (SAS Institute, USA) will be used to perform statistical analyses. Baseline characteristics will be presented as percentages (binary data), median and interquartile range (ordinal data), and mean and standard deviation (continuous data). The primary efficacy endpoint is the mean difference in the patients’ rating of patient-physician interaction (PSQ-5) between the intervention group and the control group after patients have been treated (final value) and will be analyzed with a linear mixed model accounting for recruiting site. Secondary and exploratory efficacy endpoints will be analyzed using (generalized and mixed) linear models. Reporting will include the estimator, 95% confidence intervals, and respective *p* values. No adjustments of *p* values due to multiplicity are planned.

### Interim analyses {21b}

No interim analyses will be performed.

### Methods for additional analyses (e.g., subgroup analyses) {20b}

Pre-planned subgroup analysis will be performed regarding the primary outcome variable in the subgroups in both study groups defined by the following criteria:

- Age
- Sex
- Socio-economic status
- Prior usage of SAA
- eHealth Literacy (eHEALS)
- Self-reported health (MEHM)
- Technical affinity (ATI-S)
- Study sites

### Methods in analysis to handle protocol non-adherence and any statistical methods to handle missing data {20c}

The primary population for analysis of efficacy will be the intention-to-treat population (ITT). In addition, a per-protocol analysis (PP) and a full analysis set (FAS) will be performed.

### Plans to give access to the full protocol, participant-level data, and statistical code {31c}

The full protocol will be available. The study database will be available in a de-identified form in an open data repository after the publication of the trial results.

## Oversight and monitoring

### Composition of the coordinating center and trial steering committee {5d}

The coordinating center is the Charité – Universitätsmedizin Berlin. It is an investigator-initiated study which means the Charité – Universitätsmedizin Berlin is responsible for funding, study design, management, analyses, interpretation of data, and publishing the data.

The project will be implemented and led by the Institute for Medical Informatics, Charité (Lead: FB, Co-Lead: MLS; MK). The scientific evaluation is led by the Institute of Health Services Research in Emergency Medicine, Charité (AS, MM, MBA, MB), together with the Institute of General Practice and Family Medicine, Charité (HN, KS, CH) and the Institute of Biometry and Clinical Epidemiology (SKP), Charité. Charité – Universitätsmedizin Berlin is responsible for data protection aspects, such as the creation and coordination of the data protection concept.

### Composition of the data monitoring committee, its role, and reporting structure {21a}

Since we consider symptom assessment applications of low-risk for patients and because efficacy is not assessed in safety-critical metrics, a data monitoring committee (DMC) is not needed.

### Adverse event reporting and harms {22}

Participants can report adverse events through a free-text field at the end of the questionnaire. In addition, MM and MB serve as contact persons for unexpected adverse events reported by patients and/or treating physicians.

### Frequency and plans for auditing trial conduct {23}

There will be no external, independent auditing during this trial. The Project Management Group and Trial Steering Group both meet biweekly to review trial conduct. The Ethics Committee only reviews the trial conduct if there are protocol amendments (see 25).

### Plans for communicating important protocol amendments to relevant parties (e.g., trial participants, ethical committees) {25}

Protocol amendments must be approved by the ethics committee of the Charité – Universitätsmedizin Berlin. Changes are communicated to the funding body. The protocol will also be updated in the clinical trial registry. Changes are communicated to all study personnel and study materials are updated.

### Dissemination plans {31a}

The results of the trial will be published in peer-reviewed open-access journals and presented at both national and international conferences.

## Discussion

The AKUSYM study is the first multi-center, randomized controlled trial to test the effect of a SAA on patient and physician-reported outcomes in acute care. We decided to provide the treating physicians with the recommendations of the app upfront to mimic integration into ED workflow. Results cannot be generalized to the setting where laypersons would use SAA at home for triage decisions or directed search for potential diagnoses of the symptoms they experience. Instead, study results could inform decision-makers on the implementation of a SAA into acute care to improve patient-physician interaction. We therefore experiment with a potential field of application for such apps.

## Trial status

The trial started recruitment in April 2022. Recruitment is planned to be completed by the end of September 2022 (30th of September). Trial recruitment will be continued until the sample size of 440 study participants will be reached or the funding period of the project elapses (31st of December 2022), whichever comes first. This study protocol is version 1.0 from the 28th of June 2022.

## Data Availability

De-identified data will be made available via an open data repository.

## Abbreviations

ASKU: Short Scale for Measuring General Self-efficacy Beliefs
ATI-S: Affinity for Technology Interaction Short Scale (ATI-S)
CCM: Campus Charité Mitte (One of four locations of the Charité – Universitätsmedizin Berlin)
CSQ-8: Client Satisfaction Questionnaire-8
CPQ: Cologne Patient Questionnaire
CVK: Campus Virchow-Klinikum (One of four locations of the Charité – Universitätsmedizin Berlin)
DCS: Decisional Conflict Scale
DRKS: Deutsches Register Klinischer Studien (German Clinical Trials Register)
eCRF: Electronic Case Report Form
ED: Emergency department
eHEALS: eHealth Literacy Scale
ICD-10: International Classification of Diseases Version 10
GCP: Good Clinical Practice
MAUQ: mHealth App Usability Questionnaire
MTS: Manchester Triage System
PSQ: Patient-Satisfaction-Questionnaire
SAA: Symptom assessment application
SUS: System Usability Scale
VAS-A: Visual analog scale-anxiety
WI-d: German version of the Whiteley Index
ZUF-8: Fragebogen zur Messung der Patientenzufriedenheit (ZUF-8) (German Version of the CSQ-8)
